# Investigation of CPD and HMDS Sample Preparation Techniques for Cervical Cells in Developing Computer-Aided Screening System Based on FE-SEM/EDX

**DOI:** 10.1155/2014/289817

**Published:** 2014-12-28

**Authors:** Yessi Jusman, Siew Cheok Ng, Noor Azuan Abu Osman

**Affiliations:** Department of Biomedical Engineering, Faculty of Engineering Building, University of Malaya, 50603 Kuala Lumpur, Malaysia

## Abstract

This paper investigated the effects of critical-point drying (CPD) and hexamethyldisilazane (HMDS) sample preparation techniques for cervical cells on field emission scanning electron microscopy and energy dispersive X-ray (FE-SEM/EDX). We investigated the visualization of cervical cell image and elemental distribution on the cervical cell for two techniques of sample preparation. Using FE-SEM/EDX, the cervical cell images are captured and the cell element compositions are extracted for both sample preparation techniques. Cervical cell image quality, elemental composition, and processing time are considered for comparison of performances. Qualitatively, FE-SEM image based on HMDS preparation technique has better image quality than CPD technique in terms of degree of spread cell on the specimen and morphologic signs of cell deteriorations (i.e., existence of plate and pellet drying artifacts and membrane blebs). Quantitatively, with mapping and line scanning EDX analysis, carbon and oxygen element compositions in HMDS technique were higher than the CPD technique in terms of weight percentages. The HMDS technique has shorter processing time than the CPD technique. The results indicate that FE-SEM imaging, elemental composition, and processing time for sample preparation with the HMDS technique were better than CPD technique for cervical cell preparation technique for developing computer-aided screening system.

## 1. Introduction

Cervical cancer is the third most commonly diagnosed cancer and the fourth leading cause of cancer death in females worldwide, accounting for 9% (529,800) of the total new cancer cases and 8% (275,100) of the total cancer deaths among females in 2008 [1]. The cervical cancer develops over a period of two to three decades, providing sufficient time for the screening for precursors. During adolescence, lesions are usually of low grade and the majority will regress back to normal spontaneously. A small proportion will continue to develop into true cancer precursors [2]. The incident and mortality related to this disease can be reduced through early detection. Many screening techniques have been developed for this purpose. However, these screening techniques are time consuming and contain possible human errors due to manual classification by experts. Therefore, many computer-aided screening systems have been developed for this problem. Due to the recent advancement of imaging technology, much progress has been developed in computer-aided screening system based on Pap smear [3], ThinPrep [4], colposcopy [5], cervigram [6], fluorescent in situ hybridization [7], and cervical cell FTIR [8].

FE-SEM/EDX is an electron microscopy and imaging tool which is currently used for science and technology applications. It can capture and scan structure in the surface of materials at the micro- or the nanoscale level whether organic (such as polymers, enzymes, cells, and membranes) or inorganic (such as ceramics, pigments, minerals, and composite materials). This matter is crucial to characterizing the material, understanding its mechanism and mode of formation, and explaining/predicting its properties and performance under a given set of environmental or load conditions. Therefore, computer-aided screening system can be developed based on the cervical cell images and analyzing elemental composition of the cervical cells.

However, sample preparation is a critical step in scanning electron microscopy imaging. Improper preparations of the organic and inorganic samples usually manifest one or both of these particular problems [9]:

- charging effect due to accumulation of electrons on the scanned area of sample,
- local radiation damage of the sample, induced by energetic electrons through different mechanisms such as decomposition, sputtering, sublimation, ionization, diffusion, or transformation.

Charging effect which leads to a degraded image and poor resolution and renders poor EDX analysis is caused by the incident beam being repelled from the investigated region. The charging effects were avoided or minimized for nonconducting materials by coating the sample with a thin conductive layer of gold, carbon, platinum, or gold-palladium. However, a relatively thick layer of gold may hide some nanoscale features of the sample surface. Furthermore, some samples, where specimens cannot be cut or broken for SEM observation, cannot be coated. This coating can also alter the appearance of the sample or hinder its reuse or analysis by other techniques (e.g., atomic force microscopy or Raman).

The high energetic and focused electron beam can cause serious local radiation damage to certain samples. The latter include organic and biological samples and certain inorganic materials such as metal sulfides. In order to cope with both problems, effective sample preparation techniques and low voltage scanning electron microscope are required to improve image quality and elemental analysis [10, 11].

Many researches used cells sample for SEM and/or FE-SEM investigation [12–15]. Imaging and analysis of fungal cells using high-resolution techniques particularly scanning electron microscopy (SEM) were reviewed in [12]. Meanwhile, chromosome topography using FE-SEM was presented with sample preparation on CPD technique [13]. Furthermore, sample preparation technique has been proposed based on methanol series dilutions for dehydration process. The technique was not using CPD for drying process but it was only by air-drying in desiccator [14].

However, SEM and/or FE-SEM techniques for investigation of cervical cell are very limited [9]. Effect of irreversible electroporation on adherent cervical cells has been studied [9]. For sample preparation, electrodes plate was adhered via carbon tape to aluminum stubs and loaded onto microscope stage. However, there might be a failure in the chemical fixing process of the cells at the end of experiment that caused damage to the membrane of most cells. The researchers did not describe the effective sample preparation techniques for the cervical cell in detail to be used as images for computer aided screening system.

Therefore, due to capability of FE-SEM and effect of cervical cancer, we evaluate two biological sample preparation techniques for efficient imaging of the ultrastructure of cervical cell. The preparation methods examined are based on CPD and HMDS techniques. The preparation techniques were evaluated based on FE-SEM image visualization, elemental composition, and processing time. The reason of the standard evaluations was because developing a computer-aided screening system needs many preventative image samples as training and testing.

## 2. Materials and Methods

### 2.1. Materials

SurePath is one of the liquid based cytology (LBC) techniques to screen cervical cell abnormality. This technique is the primary technique to screen for cervical precancerous cell approved by the US FDA. This procedure first scraps the cervical cell from the cervix by spatula or brush. Then, the spatula or the brush is inserted into the SurePath vial directly. In the SurePath vial, the cervical cell is suspended in fixative solution, so that it is not damaged.

**Figure 1:**
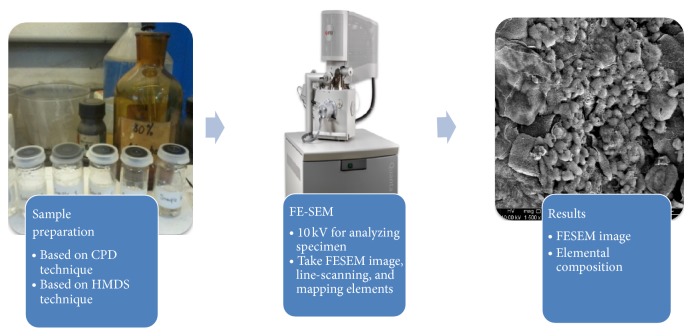
Overview of FE-SEM/EDX analysis.

In this research, SurePath specimens were collected from Gribbles Pathology laboratory, Petaling Jaya, Selangor, Malaysia. The cervical cells were obtained from 6 patients (3 normal and 3 abnormal cell samples) undergoing cervical screening for cervical precancerous cells. These samples were then sent to various laboratories in the Faculty of Dentistry, University of Malaya, Kuala Lumpur, for sample preparation and capturing image process using FE-SEM as illustrated in Figure 1.

**Figure 2:**
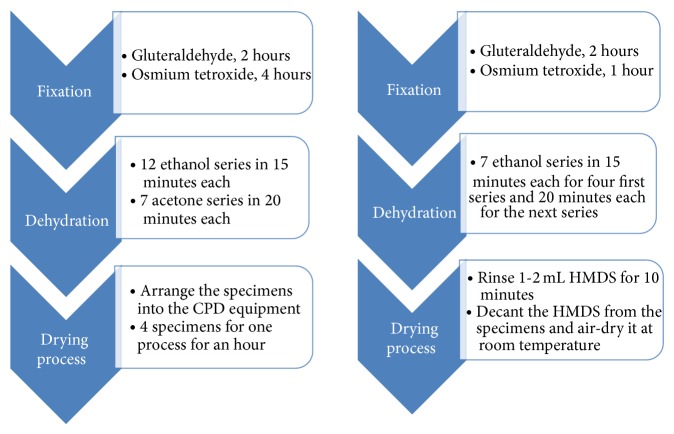
Comparison of sample preparation techniques based on CPD equipment and chemical HMDS.

In principle, sample preparation for biological specimen requires three major steps (i.e., fixation, dehydration, and drying process) as presented in Figure 2. Chemically fixed material needs first to be washed using certain technique to avoid damage of the fine structures due to surface tension. Dehydration process also requires some techniques to remove water content naturally. After dehydration process, the drying process is required to make sure of the specimen dryness and losing water molecules. In order to be scanned with an SEM and/or FE-SEM, the objects are first made conductive. This is done by coating them with an extremely thin layer (1.5–3.0 nm) of gold or gold-palladium and saving them in desiccator all times. For the cervical cell in this study, two types (i.e., CPD and HMDS) of sample preparation techniques were investigated in which the techniques have been compared for porcine retina [16]. The sample preparation processes are presented in Figure 2 and described for both techniques in detail in the subsection below.

### 2.2. Sample Preparation Technique Based on CPD

In SurePath vial, cervical cells were suspended in SurePath liquid fixation. The cervical samples were centrifuged in 4°C with 15,000 rpm to obtain the cell samples. The cells were given a drop approximately 0.1 mL on Whatman membrane (filter paper) with 13 mm 5 micron to be a specimen. For the fixation process, the specimens were rinsed twice with glutaraldehyde and osmium as presented in Figure 2. Firstly the specimens were rinsed with glutaraldehyde for 2 hours and then washed twice with 0.1% in phosphate-buffered saline (PBS) for 10 minutes each. In the second fixation, the washed specimens were rinsed with 0.1% osmium tetroxide in PBS for 4 hours at in 4°C and then washed twice with deionized water for 15 minutes each to ensure the osmium is removed from the specimens.

For the dehydration process, the specimens subsequently are dehydrated in a series of ethanol dilutions (10%, 20%, 30%, 40%, 50%, 60%, 70%, 80%, 90%, 95%, and 2x 100% dried on specimen bottle with an equilibration step of 15 minutes each) and acetone dilutions (25%, 50%, and 75%, with 20 minutes each, and 3x 100% acetone with 20 minutes each). These processes were done by 12 ethanol series and 7 acetone series.

After the dehydration process, the specimens were dried based on physical dehydration by using CPD technique with one process for 4 specimens for 1 hour, mounted on circular stainless steel moulds, coated with 10 nm of pure gold in a vacuum sputter coater, and kept in a desiccator or under vacuum to minimize artifacts caused by rehydration of the tissues from native humidity before FE-SEM/EDX data were taken using Quanta FEG 250.

### 2.3. Sample Preparation Technique Based on HMDS

For preparing the cervical cell specimen based on the HMDS, first, the two fixation processes were implemented by rinsing the specimen with 5% glutaraldehyde in 0.1% PBS for 2 hours and osmium tetroxide for 1 hour as presented in Figure 2. Between the two fixation processes, the specimen was washed three times with 0.1% in PBS for 10 minutes each. Then, after the second fixation, the specimen was rinsed twice with deionized water for 10 minutes each.

For the dehydration process, ethanol dilution dehydration series were implemented as 50%, 75%, twice with 95% with 15 minutes each, and 3 times for 100% ethanol with an equilibration step of 20 minutes each. These processes were done only by 7 ethanol series.

In the drying process, the dehydrate specimens were immersed with 1-2 mL of HMDS for 10 minutes; then decant the HMDS from the specimen vials and leave the specimen vials with the specimen in the desiccator to air-dry at room temperature. The final process was to mount the dried specimen on circular stainless steel moulds, coated with 10 nm of pure gold in a vacuum sputter coater and kept in a desiccator or under vacuum at all times before FE-SEM/EDX data were taken using Quanta FEG 250.

### 2.4. FE-SEM

**Figure 3:**
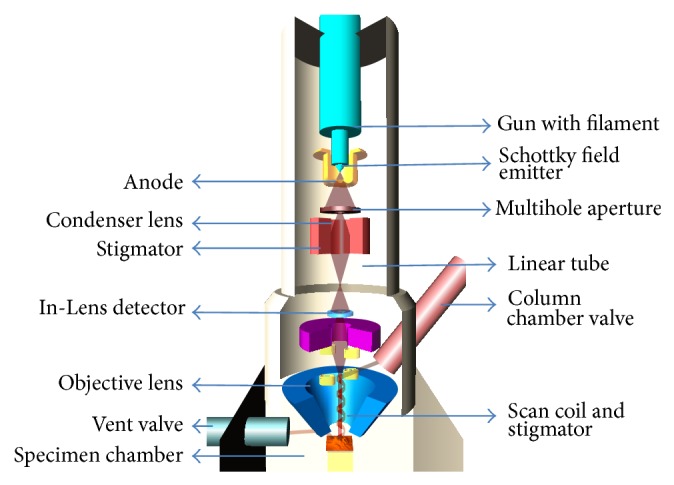
Principle of FE-SEM operations.

For principle operation as presented in Figure 3, FE-SEM uses a focused beam of electrons to generate an image or to analyze the specimen. For operation, the gun head, the column, and specimen chamber have to be evacuated. The prevacuum pump and turbo pump evacuate the specimen chamber. Vacuum in the specimen chamber is measured by penning gauge. Column chamber valve closes and N_2_ gas flows into the specimen chamber through vent valve. Schottky emitter emits electrons. The beam of electrons passes through the multihole aperture. Stigmator makes sure that the beam is rotationally symmetrical. Anode and linear tube are connected to form the beam booster. Beam booster provides better protection against external stray fields. Condenser lens controls the amount of demagnification. Objective lens focuses the electron beam onto the specimen. Deflection system consists of a set of scan coils to move the electron beam in a point-to-point scan process. In this study, FE-SEM with brand Quanta field emission gun (FEG) 250 SEM system provides flexibility and versatility to handle the challenges of today's wide ranging research needs. In both sample preparation techniques, capturing FE-SEM imaging was implemented in the same working distance (10 mm) to produce optimal imaging condition and this distance is useful for average voltage range (5 to 20 kV). Since the overarching goal of the study was to investigate the sample preparation techniques for biological samples as well as cervical cell samples to achieve high-resolution images at high magnifications, the FE-SEM was operated at low voltage (10 to 20 kV for cervical cell samples). Both In-Lens (I-L) and Everhart-Thornley (E-T) detectors were used to image the samples.

### 2.5. Techniques for Analysis

**Figure 4:**
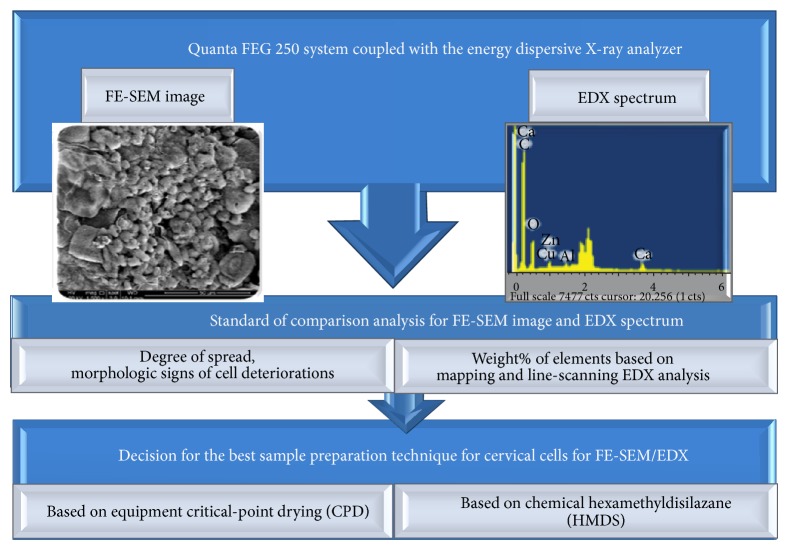
Analysis techniques for FE-SEM/EDX data based on CPD and HMDS preparation techniques.

The analyses for comparison of a better sample preparation technique for cervical cell on FE-SEM/EDX are presented in Figure 4. The sample was subjected to FE-SEM and EDX, in order to examine its localized morphology and elemental distributions at the microscopic scale. After capturing images and scanning the specimen by FE-SEM/EDX, the outputs were presented by FE-SEM images and EDX spectrum of elemental composition in the cervical cell. Analysis for the FE-SEM images of both sample preparation techniques was compared based on their degree of spread by phase contrast microscopy at 1000–5000x magnification and morphologic signs of cell deteriorations (plate and pellet drying artifacts, membrane blebs, and cytoplasmic vacuoles) [17].

Meanwhile, the EDX data were obtained using microanalytical unit that featured the ability to detect the small variations in trace element content. For EDX analysis, an accelerating voltage of 10~20 kV was used with scan time of 100 s per sampling area. Area used for EDX mapping and line EDX scanning analyses corresponded directly to the area and diameter of single cervical cell morphology examination, respectively, at 10~20kx magnification. Area of EDX mapping was subjected to a cell in one sample. For line-scanning analysis, at least ten subject cells were analyzed for the given depth of each sample. Elements chosen for analysis were based on the known chemical components of the cervical cells. The analysis of elemental distribution was comparison of an element composition in concentration and weight percentages.

## 3. Results

**Figure 5:**
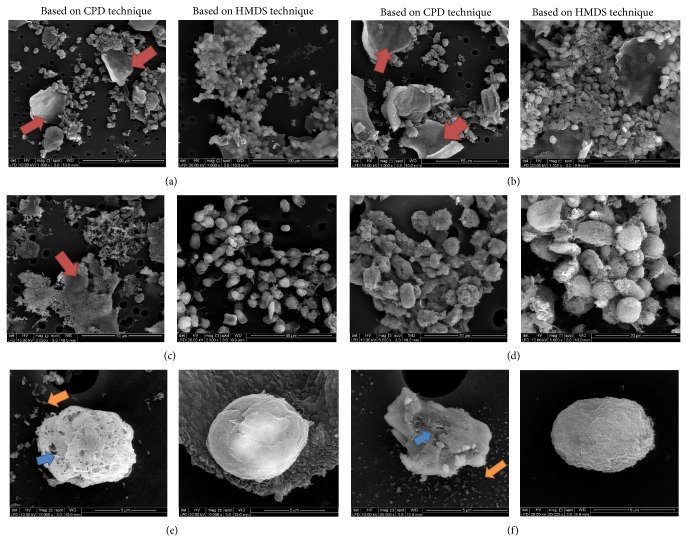
Comparison FE-SEM images of sample preparation based on CPD and HMDS techniques in different magnification; the red arrows refer to drying plate artifacts, the orange arrows refer to pellet artifacts, and the blue arrows refer to membrane blebs. (a) 1000x magnification. (b) 1500x magnification. (c) 2000x magnification. (d) 5000x magnification. (e) 15000x magnification. (f) 20000x magnification.

In this section, both outputs of capturing image and scanning EDX were presented for qualitative and quantitative analysis, respectively. The FE-SEM images from sample preparation based on CPD and HMDS techniques were compared in different magnifications as presented in Figure 5. The comparison was presented in 1kx, 1,5kx, 2kx, 5kx, 15kx, and 20kx. For 1–5kx magnifications, the degrees of cell spread in images were presented from specimens based on both sample preparation techniques while morphologic sign of cell deterioration was presented from image with 15kx and 20kx magnifications. The scanning EDX results from both CPD and HMDS techniques were presented by using area mapping and line-scanning. The composition of elemental distribution was compared in terms of weight percentages.

### 3.1. FE-SEM Imaging

FE-SEM images from the used samples were chosen to compare the effects of imaging at ever increasing magnification ranges between the CPD and HMDS techniques as presented in Figure 5. When compared to the CPD based FE-SEM images, HMDS based FE-SEM images have higher degree of spread at lower magnification (1kx–5kx magnifications). At 1kx, 1,5kx, 2kx, and 5kx, the specimens prepared by using HMDS technique were relatively full of cells as presented in Figures 5(a) to 5(d). Meanwhile, the morphologic signs of cell deteriorations which are drying plate artifacts were also presented at 1kx, 1,5kx, and 2kx presented in Figures 5(a) to 5(c). The drying plate artifacts (red arrow) were presented in more quantities on the specimens by CPD technique as compared with the specimens by the HMDS technique. Other morphologic signs of cell deteriorations were presented at 15kx and 20kx magnifications. The membrane blebs were increased in the specimen prepared by using CPD technique as pointed out by the blue arrows in Figures 5(e) and 5(f). The pellet artifacts were also raised as pointed out by the orange arrows on the specimen by using CPD technique as presented in Figures 5(e) and 5(f).

**Figure 6:**
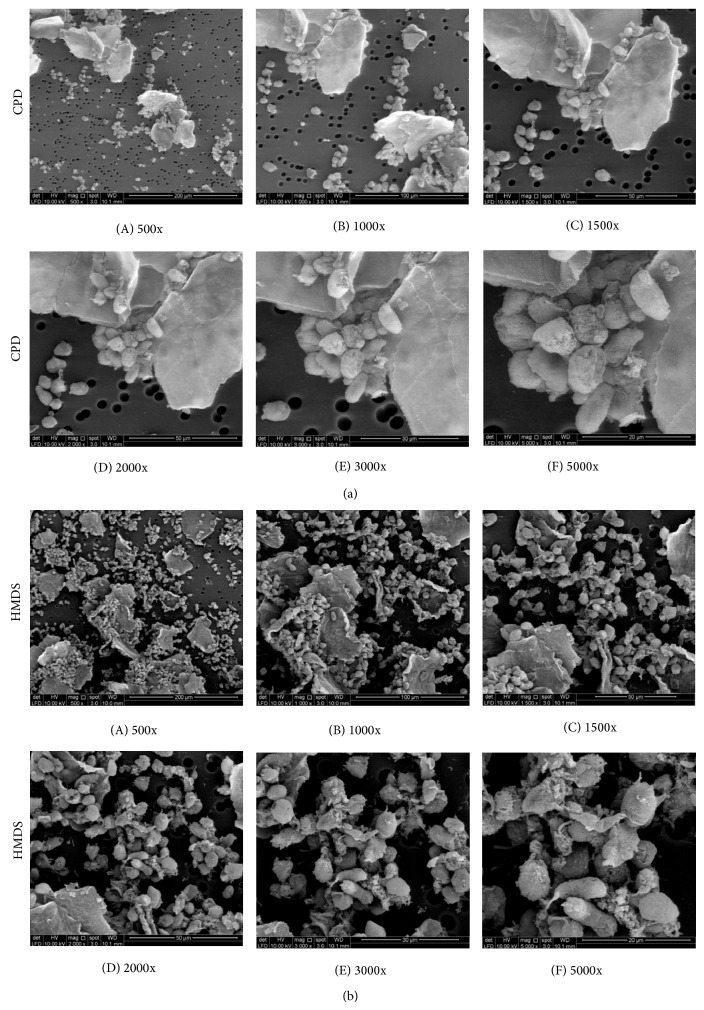
FE-SEM images of sample “a” preparation based on CPD and HMDS techniques in different magnification levels. (aA) 500x magnification, (aB) 1000x magnification, (aC) 1500x magnification, (aD) 2000x magnification, (aE) 3000x magnification, and (aF) 5000x magnification based on CPD technique and then (bA) 500x magnification, (bB) 1000x magnification, (bC) 1500x magnification, (bD) 2000x magnification, (bE) 3000x magnification, and (bF) 5000x magnification based on HMDS technique.

Furthermore, FE-SEM images in different magnification in the same area from sample “a” based on CPD and HMDS techniques were presented in Figure 6. Visually, the HMDS technique has presented higher degree of cell spread than the CPD technique as described in images with 500–1500x magnification levels. The membrane blebs had presented in higher effect for cell with the CPD technique when it was compared with the HMDS technique. The membrane blebs presented clearly in images with 2000–5000x magnification levels. Meanwhile, both techniques also have presented plate artifacts.

### 3.2. EDX Mapping and Line-Scanning

**Figure 7:**
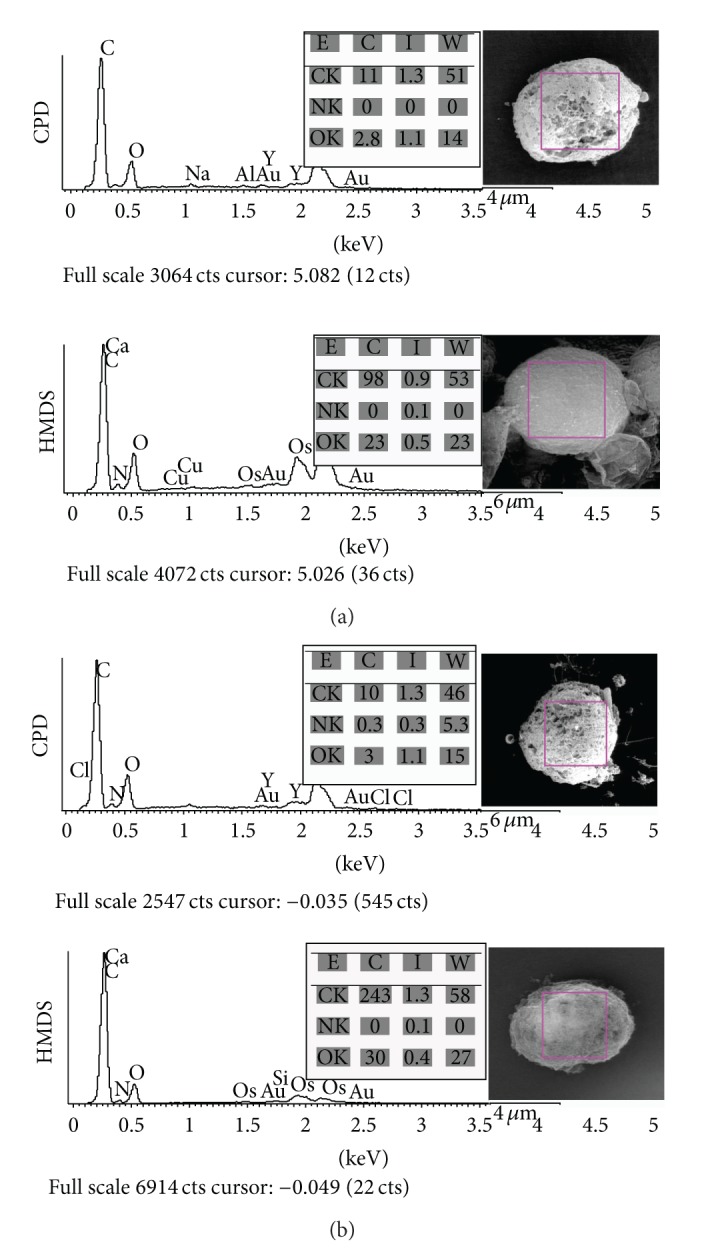
Comparison of mapping elemental analysis for sample preparation based on CPD and HMDS techniques for sample (a) and sample (b), individually. Note: E = element; C = concentration; I = intensity; W = weight; CK = carbon; NK = nitrogen; OK = oxygen.

Cervical cell samples have significant element distributions as found in this experiment. Cell area mapping results for both CPD and HMDS detected carbon (C), nitrogen (N), oxygen (O), natrium (Na), aluminium (Al), silicon (Si), and calcium (Ca) as presented in Figure 7. For elemental mapping analysis as presented in Figure 7, the sample (a) and sample (b) which were prepared based on HMDS technique had relatively higher carbon and oxygen elements than samples prepared based on the CPD technique in terms of concentration and weight percentages. Based on Figure 7, the nitrogen element was presented in very small portion and sometimes it was not detected. In sample (a) case, nitrogen element was relatively small portion detected in the HMDS technique but it was not detected in the CPD technique. Meanwhile, in sample (b) case, small portion of the nitrogen element can be detected in both techniques in terms of concentration, intensity, and weight percentage. Therefore, the nitrogen element was not then considered in this study.

Furthermore, the mapping EDX analyses were picked up for chosen six cervical cell specimens. The element contents of the mapping were distributed in different composition. As tabulated in Table 1, overall carbon and oxygen elements were significantly higher in the specimen prepared based on the HMDS technique than on the CPD technique. In Table 1, calcium and silicon elements were detected more significantly in the HMDS technique while natrium elements were detected more significantly in the CPD technique among six specimens. However, the content of the elements (i.e., calcium, silicon, natrium, and aluminium) was presented in small portion when compared with the carbon and oxygen element compositions. Meanwhile, there are certain elements not detected named as “nd” for each specimen as tabulated in Table 1. Similarly, no nitrogen level was detected among six specimens in the mapping duration time for this work.

The carbon and oxygen were then analyzed quantitatively in this study. Both elements are the organic elements in cells that were detected at low energy voltages.  Table 2 presented the comparison of the carbon and oxygen elements for the CPD and HMDS techniques in terms of weight percentage and concentration (intensity). Based on Table 2, the carbon and oxygen element compositions for all specimens prepared with the HMDS technique are significantly higher than carbon and oxygen element compositions in the CPD technique in terms of weight percentage and concentration. The carbon and oxygen element compositions were presented in bold text in Table 2. For example, for specimen 1, the elemental composition for weight percentage shows 45.18 and 21.19 to present carbon and oxygen, respectively, of the CPD technique result, while 53.76 and 23.36 to represent carbon and oxygen of HMDS technique result. Then, the concentration shows 9.31 and 4.27 (cps) to present carbon and oxygen concentration of the CPD technique results, while carbon and oxygen concentrations are presented by 103.61 and 23.95 for the HMDS technique results, whereas specimen 4 in Table 1 presented the fact that the HMDS technique is better than the CPD technique in terms of the elemental composition and concentration of both carbon and oxygen. Even though the carbon concentration of specimen 4 using the HMDS technique are specifically the lowest among the other five specimens using the HMDS technique as shown in Table 2. However, the concentration value still appears higher than the CPD technique for the specimen. It is also not so far different with specimen 1. Thus, if we see Table 2, each specimen processed by using HMDS technique has significant higher weight and concentration results of the carbon and oxygen element distribution than carbon and oxygen element compositions in the CPD technique.

For further detail analysis, the line-scanning EDX of ten cells for each sample was analyzed. The line-scanning was put in the body of cell dividing a cell to be two partitions. The line scale was the same as diameter of cells. Based on the line-scanning EDX spectra, the HMDS and CPD techniques had detected elemental distributions on the line scanning area. The carbon and oxygen element distributions were assigned different composition for each technique as well as mapping results as tabulated in Table 2.  Table 3 presented comparison of line-scanning EDX for carbon and oxygen elements of 10 captured cells for each specimen based on CPD and HMDS techniques in terms of weight percentage. As presented in Table 3, the average carbon elements of 10 captured cells for each specimen prepared with HMDS technique were significantly higher than the average carbon elements with CPD technique which are proved by ANOVA statistical analysis with* P* value < 0.05. The average oxygen elements of the specimens prepared with HMDS technique were relatively higher than specimens prepared with CPD technique. From Table 3, overall, the average of carbon and oxygen element compositions for each specimen based on HMDS technique was relatively higher than the average element compositions based on CPD technique.

## 4. Discussion

In this study, CPD and HMDS preparation techniques can provide morphological image and elemental spectrum for cervical cells. Based on the Results section, the HMDS produced better image quality and elemental composition than the CPD technique. The reasons of the obtained results were discussed in this section. Although CPD was the most common preparation technique, HMDS required no specialized equipment and no precise monitoring of the samples, resulting in lower time and cost commitments than CPD technique.

Based on the FE-SEM image of cervical cells presented in Figures 5(a) to 5(d), the cervical cells presented higher degree of spread for the specimens prepared using HMDS technique. The specimen prepared based on HMDS technique kept many cells compared with the CPD technique. This condition was because the CPD technique had been done by 12 ethanol series and 7 acetone series. The cells which stick on the membrane filter were considered loose during the change of series. Therefore, these multiseries can affect reduction of cells. In contrast, compared with the HMDS preparation technique, the technique had only been done by 7 ethanol series in the fixation process. Hence, many cells still exist and adhere to the membrane filter. In addition, the CPD technique for drying process was generally considered essential for the preparation of biologic specimens for electron microscopy. However, as usually carried out it has the disadvantage that the initial flow of gas from the cylinder may blow away minute specimens such as cells on the membrane filter [18, 19].

Signs of cell deterioration as presented as visual analysis were membrane bleb, plate, and pellet drying artifact presented in the Results section for FE-SEM cell image. The membrane blebs on cell could be seen in 15kx and 20kx magnification on individual cell. Based on [14], the membrane blebs could be also caused by the fixation with osmium tetroxide for 4 hours (categorized long for cell samples). Blow-up of cell's membrane due to the osmium tetroxide can produce the small pellet as part of the cells. After the dehydration process, the pellet could dry and then appear as pellet drying artifacts in capturing images. Meanwhile, the plate drying artifact presented in Figures 5(a) to 5(c) was also caused by fixation process on the CPD technique. The artifacts were signs of sample disruptions which can occur with osmium tetroxide fixed specimen [14]. The CPD technique used the osmium fixation for four hours which is longer than HMDS technique. Therefore, the HMDS technique for sample preparation has better image results than the images resulting from the CPD sample preparation technique.

Furthermore, based on the mapping and line-scanning EDX results, carbon and oxygen were significant elements existing in the cervical cells. The other elements appeared but in small portion of intensity as presented in Table 1. When excited by electrons of sufficient energy, every element in a sample will emit a unique and characteristic pattern of X-rays. Furthermore, under given analysis conditions in this study (accelerating voltage and duration time for scanning process) the number of X-rays emitted by each element bears a more or less direct relationship to the concentration of that element [10, 20]. These X-ray emissions then were converted to analyzable data as described in Figure 7 by a series of electronic components. As presented in Figure 7 and Table 1, besides carbon and oxygen elements, the other elements appeared in small portion of intensities. The relationship of the intensity was addressed to the concentration and then percentage of weight. However, the elements appeared in very small portion due to the duration time of scanning and content in the cell. Therefore, the other elements were not considered in this paper.

By comparing carbon and oxygen results for both techniques as presented in Tables 2 and 3 the HMDS techniques had significantly higher element compositions than the CPD technique. This effect was caused by the CPD technique which used dehydrants like ethanol and acetone in 19 series. Dehydration always results in shrinkage as water molecules are removed from the cell structure. However, ethanol or acetone cannot be performed in long series as suggested in previous study using stem cells [14]. These dehydrants may dissolve the lipid content in cells, followed by transitional fluids such as liquid CO_2_ and freons for drying the specimen [14]. In contrast, the acetone series solutions did not exist and the ethanol series were applied only in 7 series by using the HMDS technique. Hence, these conditions affected level composition of carbon and oxygen elements in the cells.

In addition, in terms of the time processing, sample preparation based on the HMDS technique was rapid and faster than the CPD technique. As explained in the methodology section, the CPD technique has 6 hours in fixation process, 19 ethanol and acetone dilutions series for 5-6 hours in dehydration process, and drying using CPD technique for 1 hour operated only in four specimens for one process of sample preparation. It was meant that only 4 specimens were obtained in 13-hour processing time. However, the preparation of samples based on the HMDS can be done by 12 or more specimens for one process. The processing time was also reduced by avoiding the osmium tetroxide after fixation for long time (4 hours), only 7 series of ethanol dehydrants, and drying process by using the chemical HMDS only done for 10 minutes. It was meant that 12 or more specimens can be obtained for 5 hours plus 10 minutes (i.e., 3 hours for fixation, 2 hours for dehydration, and 10 minutes for drying processes). Therefore, the CPD technique was avoided to be used as sample preparation technique for cervical cell in order to be employed for computer-aided screening system study.

Since FE-SEM analysis is a simpler method to validate the morphological data and the elemental distribution from EDX mapping and line-scanning could be features to differentiate the cervical cell classes, studies on how classification system based FE-SEM/EDX features can be challenged are performed in further study. Thus, we have recommended a sample preparation technique for cervical cell, namely, HMDS technique. By using seven series of ethanol only as dehydrants, avoiding the long time use of toxic osmium tetroxide for postfixation, and not using CPD, we found a better technique for preparation of cervical cells for FE-SEM/EDX analysis which has better image quality, higher elemental composition, and rapid process and is safer and cost effective.

## 5. Conclusion

This work investigated the efficient sample preparation technique for cervical cell which can be used for sample preparation for developed computer-aided screening system for cervical cell based on FE-SEM image and EDX spectrum. For the conclusion, we have recommended a sample preparation technique for cervical cell by using HMDS technique. It was found as a better technique for preparation of cervical cells for FE-SEM/EDX analysis which is safer and rapid and cost effective.

## Figures and Tables

**Table 1:** Elemental composition of mapping EDX of six cervical samples based on CPD and HMDS techniques.

Preparation techniques	Name of specimens	Elemental composition (weight %)					
		C	O	Al	Na	Ca	Si
Based on CPD technique	*Specimen1 *	45.18	21.19	0.39	0.96	1.5	nd
	*Specimen2 *	56.19	17.52	nd	0.43	0.57	nd
	*Specimen3 *	35.35	19.09	4.81	0.29	3.39	5.66
	*Specimen4 *	43.1	18.46	0.67	nd	nd	1.64
	*Specimen5 *	46.4	14.93	nd	nd	nd	nd
	*Specimen6 *	45.81	13.47	nd	0.53	nd	nd

Based on HMDS technique	*Specimen1 *	53.76	23.36	0.19	nd	1.18	nd
	*Specimen2 *	71.63	23.53	0.37	0.41	0.45	1.83
	*Specimen3 *	63.9	19.4	nd	nd	0.41	nd
	*Specimen4 *	46.08	41.31	4.77	0.14	0.3	5.39
	*Specimen5 *	67.83	26.62	nd	nd	0.51	0.21
	*Specimen6 *	67.81	15.35	nd	nd	nd	nd

**Table 2:** Comparison of carbon and oxygen elements based on CPD and HMDS sample preparation techniques in terms of concentration and weight percentage for each specimen.

Name of specimens	Elemental composition (weight %)				Concentration (cps)			
	CPD		HMDS		CPD		HMDS	
	C	O	C	O	C	O	C	O
*Specimen1 *	45.18	21.19	**53.76**	**23.36**	9.31	4.27	**103.61**	**23.95**
*Specimen2 *	56.19	17.52	**71.63**	**23.53**	13.35	3.31	**283.5**	**29.82**
*Specimen3 *	35.35	19.09	**63.9**	**19.4**	6.45	4.6	**156.31**	**19.3**
*Specimen4 *	43.1	18.46	**46.08**	**41.31**	9.93	3.65	**96.89**	**73.66**
*Specimen5 *	46.4	14.93	**67.83**	**26.62**	10.42	2.97	**242.59**	**30.32**
*Specimen6 *	45.81	13.47	**67.81**	**15.35**	10.44	2.69	**153.2**	**13.79**

**Table 3:** Comparison of line-scanning EDX for carbon and oxygen elements of 10 captured cells for each specimen based on CPD and HMDS techniques in terms of %weight.

	Carbon elements		Oxygen elements	
	CPD	HMDS	CPD	HMDS
*Specimen1 *	36.51 ± 6.59	56.35 ± 6.08	22.56 ± 2.08	29.69 ± 4.63
*Specimen2 *	42.95 ± 6.17	63.31 ± 10.07	20.37 ± 4.98	29.78 ± 5.09
*Specimen3 *	35.60 ± 2.59	48.17 ± 13.30	25.31 ± 2.03	26.83 ± 6.22
*Specimen4 *	39.22 ± 4.47	58.93 ± 11.09	23.29 ± 3.82	31.15 ± 6.40
*Specimen5 *	48.23 ± 1.95	70.14 ± 2.58	13.97 ± 0.68	25.65 ± 1.89
*Specimen6 *	49.34 ± 1.47	62.80 ± 2.18	13.27 ± 0.57	18.96 ± 1.23

^*^ *P* value for each specimen shows not more than 0.05.
